# Electrical Impedance Spectroscopy with Bacterial Biofilms: Neuronal-like Behavior

**DOI:** 10.1021/acs.nanolett.3c04446

**Published:** 2024-02-06

**Authors:** Emmanuel U. Akabuogu, Lin Zhang, Rok Krašovec, Ian S. Roberts, Thomas A. Waigh

**Affiliations:** † Division of Infection, Lydia Becker Institute of Immunology and Inflammation, School of Biological Sciences, University of Manchester, Oxford Road, Manchester M13 9PT, United Kingdom; ‡ Biological Physics, Department of Physics and Astronomy, University of Manchester, Oxford Road, Manchester M13 9PL, United Kingdom; § Division of Evolution, Infection and Genomics, School of Biological Sciences, Faculty of Biology, Medicine and Health, University of Manchester, Manchester M13 9PT, United Kingdom; ∥ Photon Science Institute, Alan Turing Building, Oxford Road, Manchester, M13 9PY, United Kingdom

**Keywords:** electrical impedance spectroscopy, neuron, bacteria, biofilm, voltage-gated ion channel, negative capacitance

## Abstract

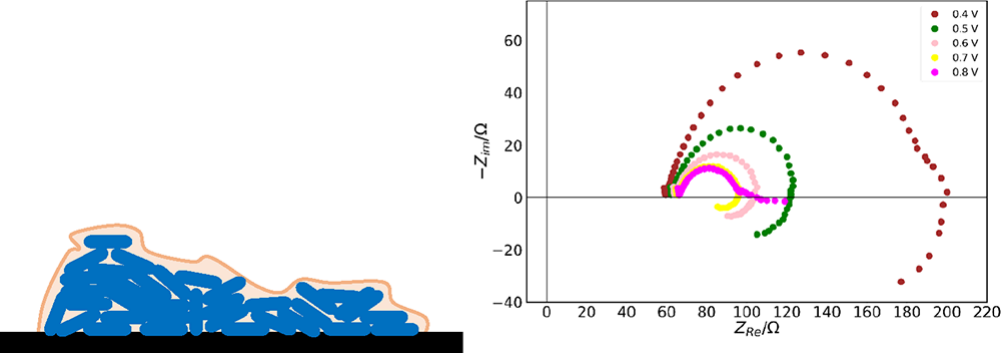

Negative capacitance at low frequencies for spiking neurons was first demonstrated in 1941 (K. S. Cole) by using extracellular electrodes. The phenomenon subsequently was explained by using the Hodgkin–Huxley model and is due to the activity of voltage-gated potassium ion channels. We show that *Escherichia coli* (*E. coli*) biofilms exhibit significant stable negative capacitances at low frequencies when they experience a small DC bias voltage in electrical impedance spectroscopy experiments. Using a frequency domain Hodgkin–Huxley model, we characterize the conditions for the emergence of this feature and demonstrate that the negative capacitance exists only in biofilms containing living cells. Furthermore, we establish the importance of the voltage-gated potassium ion channel, Kch, using knock-down mutants. The experiments provide further evidence for voltage-gated ion channels in *E. coli* and a new, low-cost method to probe biofilm electrophysiology, e.g., to understand the efficacy of antibiotics. We expect that the majority of bacterial biofilms will demonstrate negative capacitances.

Transport of ions through voltage-gated ion channels in cellular membranes produces spiking potentials in neurons.^1,2^ Historically, the electrical stimulation of neurons was investigated by Cole (1941), who was surprised to observe a large negative capacitance in electrical impedance spectroscopy experiments at low frequencies. The negative capacitance was explained using calculations based on the Hodgkin–Huxley model.^3,4^ It is directly related to the spiking phenomenon and the activity of the voltage-gated potassium ion channels.^3−6^

Antibiotic-resistant bacteria currently present a huge problem to mankind.^7,8^ Bacteria embedded in biofilms can be resistant to 2–3 orders of magnitude higher concentrations of antibiotics, contributing to the problem.^9^

Electrical impedance spectroscopy has been previously applied to bacteria and bacterial biofilms.^10−12^ However, the electrical impedance spectra looked similar to other organic layers, and no specific neuronal-like behavior has previously been observed, e.g., spiking potentials or negative capacitances.

Theoretical analysis shows the negative capacitance of neurons is crucial for their nonlinear dynamics including spiking, bifurcations and stability phenomena.^13^ Negative capacitance also exists in inorganic systems, such as electrochemical cells,^14^ memristors, and solar cells.^15−17^ For such systems, it can control catalytic processes, corrosion, memory effects, and electrodeposition. In neurons, spiking potentials are caused by fluxes of Na^+^ and K^+^ ions through voltage-gated channels in the cell membranes.^1,2^ Knowledge of this mechanism of neuronal dynamics has inspired synthetic devices that imitate spiking potentials in neurons. A specific example is the memristor, which has voltage- and time-dependent resistance and negative capacitance.^18,19^ Memristors can adjust their resistance at a set voltage to mimic the spiking potential of neurons for neuromorphic computing. Memristors undergo resistive switching due to the amount of current that previously flowed, so they can act as memory devices.^20,21^

Biofilms are colonies of bacteria living together in extracellular polymeric substance (EPS) adhered to surfaces.^9,22^ Bacteria in biofilms can have membrane potential spiking similar to neurons,^23−30^ although the hyperpolarization phenomena tend to be more varied than the stereotyped spiking events observed in neuronal cells. In the presence of external stimuli, such as metabolic stress,^24^ photooxidative stress,^25,30^ biosurfactants,^31^ and external electrical stress,^32^ cells in biofilms open their voltage-gated channels to allow the flow of cationic ions. In *Bacillus subtilis* (*B. subtilis*) biofilms, the *YugO* channel controls the flux of potassium ions and is implicated in electrical signaling.^24−26,28,33,34^*Escherichia coli* (*E. coli*) cells and biofilms engage in membrane potential spikes when stimulated by an electric field, although electrophoresis of fluorophores can complicate experiments.^32,35,36^ Our previous work also shows that the voltage-gated Kch channels in *E. coli* biofilms cause potassium ions to flow when exposed to light stress.^23^ The flow of these ions triggers phases of hyperpolarization and repolarization, which lead to electrical signaling across the biofilm. Neighboring cells in biofilms serve as conduits that propagate a wave of spikes via a fire–diffuse–fire mechanism.

In neuronal systems the effect of negative capacitance can be studied both in the time and frequency domains.^37^ The time-domain version of the Hodgkin–Huxley (HH) model^6^ can be converted to the frequency domain using Laplace transforms.^3,5^ Equivalent circuits can be used to model the impedance spectra of samples under an external voltage stimulation. The frequency-domain version of the HH model aids the study of ion channels and spike mechanisms, since it automatically averages over many thousands of stimulation events, providing succinct information not available with time-domain analysis (e.g., their dynamic phase behavior) and improved signal-to-noise. The frequency domain impedance response of the HH model for the spiking squid axon under small amplitude perturbation was originally calculated by Cole^3,4^ and the analysis was recently extended by Bou and Bisquert.^5^

Electrochemical impedance spectroscopy (EIS) is a key experimental technique to probe the frequency-domain electrical responses of electrochemical systems.^38,39^ In EIS, small-amplitude sinusoidal electrical perturbations are applied to thin films attached to electrodes and the frequency is varied over many decades. Analysis of the resulting complex impedance produces information about the electrochemical dynamics. The technique can also be used to decipher the spiking dynamics of neurons to voltage stimulation.^5^

We characterized the impedance spectra of *E. coli* biofilms. We created an equivalent circuit (EC) model and subsequently established the frequency response of *E. coli* biofilms to small-amplitude voltage perturbations.

Kch, a potassium ion channel in *E. coli*, was discovered (Milkman in 1994) using comparative genetics techniques.^40^ Its exact physiological role in *E. coli* is not completely understood, although there is circumstantial evidence that it is voltage-gated^41^ and previous evidence from our group shows that it is involved in electrosignaling in response to light stress.^23^

We used the method of P1 phage transduction to genetically move the Kch mutation from *E. coli* K-12 BW25113 Δ*kch* mutant, a strain lacking the *kch* gene, into wild-type (WT) DH5α. We confirmed the *E. coli* DH5α Δ*kch* mutant phenotype using appropriate PCR primers. Biofilms of the wild-type (WT) DH5α and DH5α Δ*kch* mutant strains were grown on ITO electrodes. DH5α grows readily into a biofilm,^42,43^ and removal of the Kch gene does not impede its growth.^23,44,45^ Both strains exhibited similar growth curves determined using a plate reader over 24 h (Figure 1a). Next, we studied biofilm EIS after 24 h growth, applying a 10 mV sinusoidal voltage over a frequency range of 2 × 10^–1^–10^5^ Hz with a 0 V DC voltage. DH5α exhibited distinct features in its Nyquist plot (Figure 1b) that were not observed in the DH5α Δ*kch* mutant (Figure 1c). Two distinct impedance arcs were observed in DH5α. The EIS spectra were stable and reproducible over the entire frequency range (Figure S2a,b). The impedance arc within the high-frequency region (Figure 1b, at the small impedances) was not seen in the DH5α Δ*kch* mutant (Figure 1c), so it must be due to the Kch ion channels. We then analyzed the contact resistance (*R*_ct_) within the biofilms. *R*_ct_ depends on the rate of exchange of charge at the electrode/biofilm interface.^46^ We designed an equivalent circuit (EC) model of the representative impedance spectra for both systems (Figure 1b inset and Figure 1c inset) and deduced the resistances *R*_ctWT_ and *R*_ctMutant_ for DH5α and the DH5α Δ*kch* mutant, respectively. Remarkably, the contact resistance of DH5α Δ*kch* mutant was *R*_ctMutant_ = 7500 ± 28 Ω, 15-fold higher than that for the wild-type DH5α (Figure 1d), *R*_ctWT_ = 471 ± 4 Ω. This suggests that the charge transfer between the electrode and the biofilm is much more efficient when Kch ion channels exist in the *E. coli* membranes.

**Figure 1:**
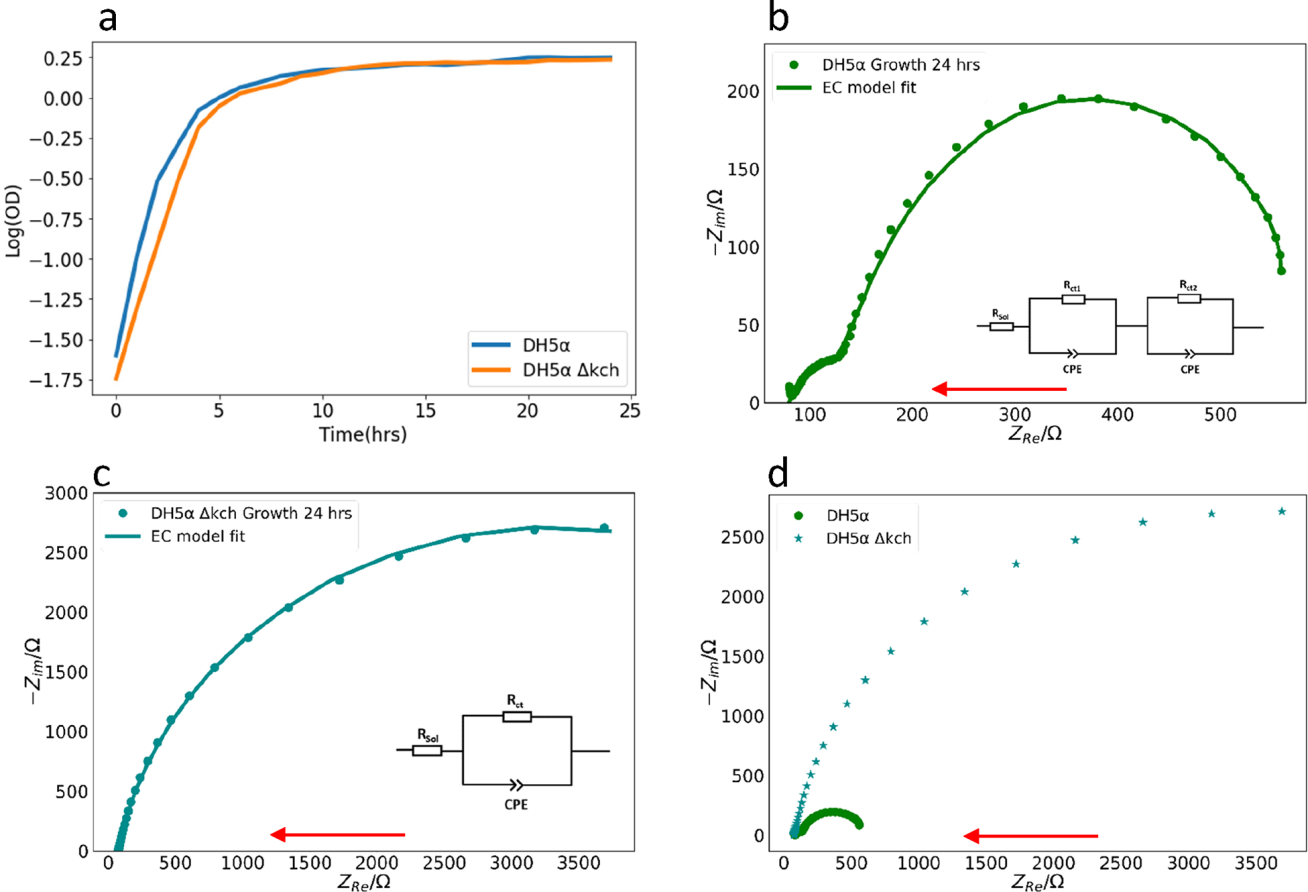
(a) Growth curve for DH5α and DH5α Δ*kch* over 24 h from a plate reader. OD is the optical density. (b) Representative impedance spectrum for DH5α showing the imaginary impedance (−*Z*_im_) plotted as a function of the real impedance (*Z*_Re_). Inset: Equivalent circuit model was used to fit the data. The solid line represents the model fit using the equivalent electric circuit (EC) shown in the inset, and the dots represent the experimental data. (c) Representative impedance spectrum for DH5α Δ*kch*. Inset: Equivalent circuit model used to fit the data. The solid line represents the model fit using the EC shown in the inset, and the dots represent the experimental data. (d) Comparison between EIS spectra of both strains obtained after the 24 h biofilm culture. All data were obtained from at least three experimental replicates. Red arrows show the direction of an increasing frequency. CPE is the constant phase element. *R*_sol_ and *R*_ct_ are the solution resistance and contact resistance of DH5α Δ*kch*, respectively. *R*_ct1_ and *R*_ct2_ are the contact resistances of wild-type DH5α biofilms.

We then monitored the growth-dependent change in EIS. We performed the EIS experiments for biofilms grown for 16–24 h. The impedance plots for both strains revealed a steady decrease (Figures 2a,b) of the impedance arc; i.e., larger more mature biofilms have lower impedances. The *R*_ct_ plot over time also confirmed a decreased contact resistance of DH5α compared to DH5α Δ*kch* (Figure 2c).

**Figure 2:**
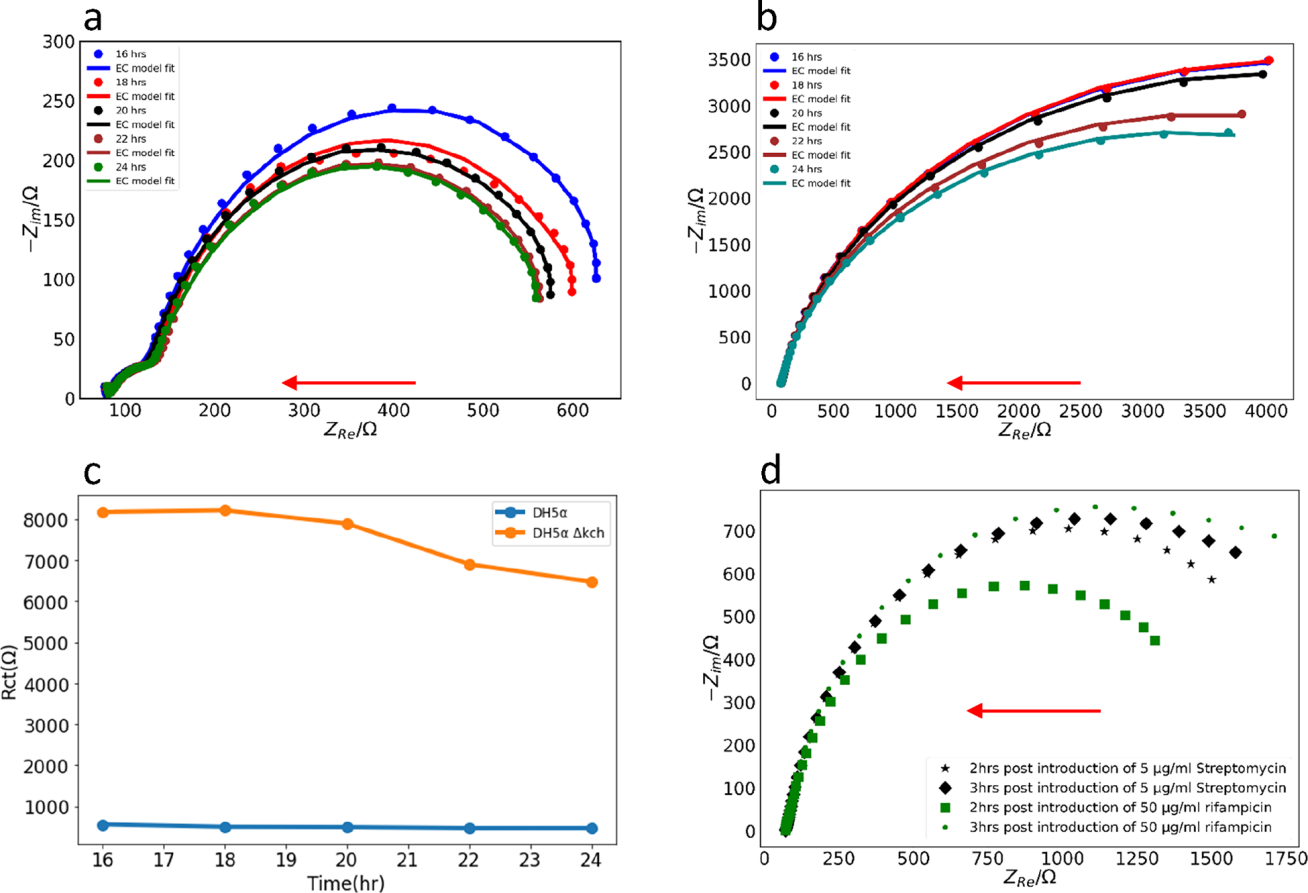
(a) Variation of EIS spectra of DH5α biofilm as a function of growth time. Solid lines represent the model fits using the EC shown in the inset of Figure 1b and dots represent the experimental data. (b) Variation of EIS spectra of DH5α Δ*kch* biofilm as a function of growth time. Solid lines represent the model fits using the EC shown in the inset of Figure 1c, and the dots represent the experimental data. (c) Time-dependent evolution of the charge transfer resistance in DH5α and DH5α Δ*kch* biofilm. For DH5α, the *R*_ct2_ from a low-impedance arc was used. (d) Antibiotic-dependent changes in the EIS spectra obtained from DH5α biofilm. Red arrows show the direction of increasing frequency.

Next, we examined the effect of antibiotics on the impedance spectra of the DH5α strain. We employed two antibiotics, rifampicin and streptomycin, at concentrations higher than their minimum inhibitory concentrations. Rifampicin (net zero charge) inhibits bacterial RNA polymerase,^47^ whereas streptomycin (positively charged) interferes with protein synthesis by ribosomes.^48^ We observed an increase in the radius of the impedance arc and the *R*_ct_, as the concentration of the antibiotics increased; i.e., charge transfer was decreased by the antibiotics. Rifampicin showed a larger time-dependent increase in the *R*_ct_ compared to streptomycin (Figure 2d).

To explore the EIS spectra of *E. coli* biofilms in more detail, we conducted experiments at a range of DC bias voltages, 0.1–0.8 V. Experiments at different DC bias voltages help to monitor time-dependent memory effects in electrochemical systems.^5,15,38,49^ When the *V*_app_ was increased from 0.1 to 0.3 V, there was a corresponding steady decrease in the radius of the impedance arc (Figure 3a). The small semicircle observed at high frequencies in the complex impedance plot at 0 V (Figure 1b and Figure 2a) due to Kch ion channels disappeared. Furthermore, at a *V*_app_ of 0.4 V, the low-frequency region becomes negative in the complex impedance plot; i.e., a negative capacitance is observed. The impedance arc in the fourth quadrant of the impedance plot remained stable and persisted until the *V*_app_ approached 0.7 V. At a *V*_app_ of 0.8 V, the impedance arc in the fourth quadrant suddenly disappears, although the enlarged arc in the first quadrant remained (Figures 3a,b). A *V*_app_ value of 0.4 V represents the threshold voltage for the appearance of negative capacitance. Further increases in the applied voltage *V*_app_ beyond 0.8 V yielded noisy data within the fourth quadrant.

**Figure 3:**
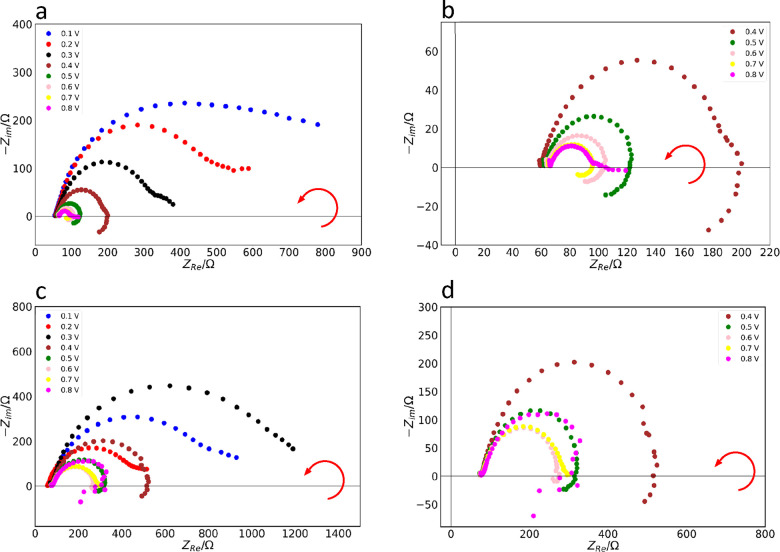
(a) Electrical impedance spectra of DH5α biofilms at voltages 0.1–0.8 V. (b) Electrical impedance spectra of DH5α at DC bias voltages 0.4–0.8 V, highlighting the negative capacitance. (c) Electrical impedance spectra of DH5α Δ*kch* at DC bias voltages 0.1–0.8 V. (d) Electrical impedance spectra of DH5α Δ*kch* at DC bias voltages 0.4–0.8 V, highlighting the negative capacitance. Red arrows show the direction of increasing frequency.

To understand the contribution of the Kch gene to the EIS data, we conducted EIS on the DH5α Δ*kch* biofilms under a variety of applied DC bias voltages. At a *V*_app_ of 0.1 V, we observed a similar-shaped spectra to the wild type (Figure 3c). The impedance arc decreased in radius at a *V*_app_ of 0.2 V but increased sharply at a *V*_app_ of 0.3 V. The impedance spectra transitioned to the fourth quadrant when the applied voltage was increased to 0.4 V; i.e., a negative capacitance was observed. Compared with the wild-type biofilm, the radius of the impedance arc in the first quadrant of DH5α Δ*kch* was larger for each *V*_app_ (lower impedances, Figure S2c) and the impedance arc formed in the fourth quadrant disappeared at a *V*_app_ of 0.7 V (Figure 3d). The negative capacitance arc existed over a wider range of applied voltages in the wild type than in the mutant (Figure 3b,d). The observed patterns of the EIS data for the DH5α and DH5α Δ*kch* biofilms at *V*_app_s of 0.4–0.7 V (Figure 3a–d) and their stability are consistent with the phenomenon of negative capacitance.^3−5,37^ The disinfectant Virkon was then added to DH5α biofilms so they predominantly contain nonviable cells, and the negative capacitance behavior was removed in EIS data (Figure S2d).

Motivated by work on the frequency domain response of the Hodgkin–Huxley (HH) model of the squid axon^4,5,50^ and our time-domain version of the HH model for *E. coli* biofilms,^23^ we developed equivalent circuits to characterize the frequency-domain impedance response of the *E. coli* biofilms under small DC bias voltages (Supporting Information). Our focus was on the electrical impedance spectra produced at applied voltages of 0.4–0.7 V for the WT and 0.4–0.6 V for the Kch mutant where the negative capacitance phenomena were observed. Figure 4a is the predicted partial equivalent circuit for a single variety of voltage-gated ion channel.^5^ The first branch contains the membrane capacitance (*C*_M_), and the second branch contains the resistance (*R*_*a*_) across *a*, a single variety of ion channel. The third branch is a parallel connection to a resistor–inductor (*R*_*x*_*L*_*x*_) circuit element, which accounts for the inductive effect that produces the negative capacitance. *R*_*x*_ is the resistance across the gating variable *x*, and *L*_*x*_ is the inductance across the gating variable *x*. The final branch consists of the resistance (*R*_l_) of the leak channel. While the partial circuit (Figure 4a) accounts for the membrane capacitance, ion conduction, and the negative capacitance effects, more circuit elements are required to fit the experimental spectra to account for charge transfer to the biofilm (Figure 3b,d). Therefore, we created a more complex minimal equivalent circuit (Figure 4b,c). In our new equivalent circuit (Figure 4b), we added additional components to the single ion channel equivalent circuit (Figure 4a) to describe the solution (*R*_sol_) and interfacial contact (*R*_ct_) contributions to the overall resistance. We also included a constant phase element (CPE) to account for heterogeneities in the electrode surface^51^ and the formation of EPS in the biofilm.^11^ The full equivalent circuit (Figure 4b) described the impedance spectra of the voltage-gated ion channel(s) responsible for the negative capacitance observed in the DH5α Δ*kch* mutant (Figure 3d) and provides a description of non-Kch voltage-gated channel(s) in the knockout mutant strain. We represent these voltage-gated ion channel(s) with the symbol *Q* and the cumulative resistance across those channels as *R*_*Q*_. *R*_*n*_ is the resistance associated with the gating variable *n* for the *Q* channel. The resistances *R*_*Q*_ and *R*_*n*_ and the time constants of the equivalent circuits were calculated as a function of the *V*_app_ (Figure 4d,e and Supporting Information Table S1).

**Figure 4:**
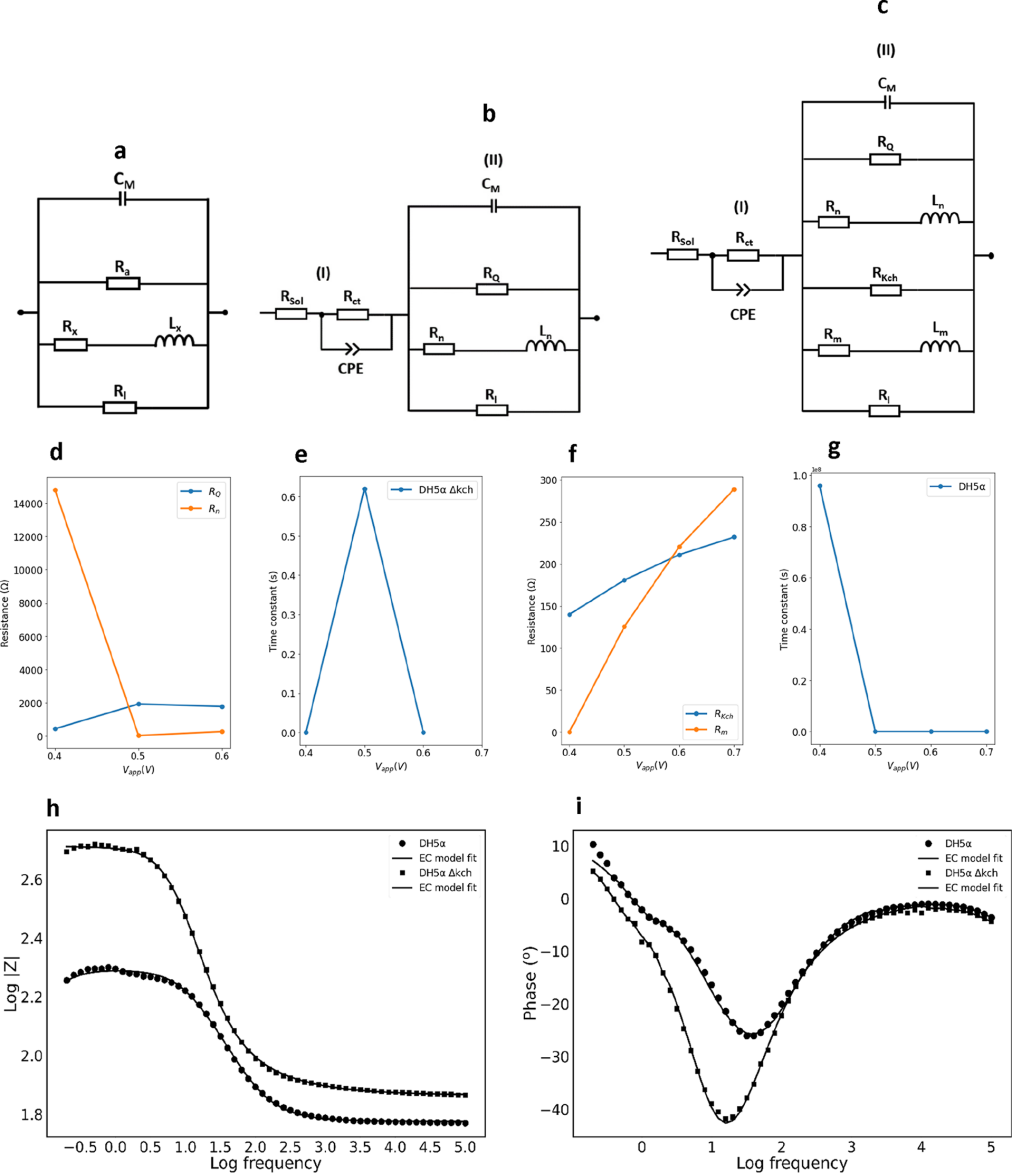
(a) Partial equivalent circuit (EC) for the frequency domain Hodgkin–Huxley model for a single variety of voltage-gated ion channel, based on information given in ref (5). (b) EC used to fit the EIS data for the *Q* channel in DH5α Δ*kch*, Figure 3d (0.4–0.6 V). (c) EC used to fit the EIS data for the Kch channel in the wild-type DH5α, Figure 3b (0.4–0.7 V). (d) Values of the resistance for the range of applied voltages for the *Q* ion channel. Data were obtained from Figure 3d using the EC fit of Figure 4b. *R*_*Q*_ and *R*_*n*_ are the resistances across the *Q* ion channel and its gating variable *n*, respectively. (e) Values of time constant for the *R*_*n*_*L*_*n*_ branch of the *Q* channel (Figure 4b) for the range of applied voltages. *L*_*n*_ is the inductance across the gating variable *n*. (f) Values of the resistance for the range of applied voltages for the Kch ion channel of DH5α. Data obtained from Figure 3b using the EC fit of Figure 4c. *R*_Kch_ and *R*_*m*_ are the resistances across the Kch ion channel and its gating variable *m*, respectively. (g) Values of the time constant for the *R*_*m*_*L*_*m*_ branch of the Kch channel (Figure 4c, Figure 3b) for the range of applied voltages. *L*_*m*_ is the inductance across the gating variable *m*. (h) Representative Bode plot (the change in the impedance modulus |*Z*| as a function of frequency) for both strains of *E. coli* at an applied DC bias of 0.4 V. Solid lines represent model fits using the EC (Figure 4c, DH5α; Figure 4b, DH5α Δ*kch*), and the dots represent the experimental data. (i) Representative Bode plot (the change in the phase as a function of frequency) for both strains at an applied DC bias of 0.4 V. Solid lines represent the model fits using the EC (Figure 4c, DH5α; Figure 4b, DH5α Δ*kch*), and the dots represent the experimental data. CPE is the constant phase element. *R*_sol_ and *R*_ct_ are the solution resistance and contact resistance, respectively.

Next, we designed a complete equivalent circuit (EC) for the wild-type DH5α biofilm (Figure 4c). This EC incorporates both the Kch channel and the other voltage-gated channels, *Q*. Data fits obtained for Kch ion channels in the WT, Figure 3b, using the circuit, Figure 4c, revealed increasing resistance values for *R*_*Kch*_ and the resistance of its gating variable, *R*_*m*_ with applied DC voltage (Figure 4f and Table S2). The time constant for the *RL* branch has a sharp drop as a function of the applied voltage, followed by a plateau (Figure 4g). We confirmed that using the complete EC (Figure 4c) to fit the DH5α Δ*kch* mutant spectra yields the same trend for *R*_*Q*_ and *R*_*n*_ (Figure S3a) as with the EC of Figure 4b; i.e., the two models are mutually compatible.

We used Bode plots (Figure 44h,i) for the dependence of the total impedance and phase on frequency. Figure 4h shows that, for both strains, the impedance modulus had a slight rise within the low-frequency region and then a sharp drop within the mid-frequency region. However, across the entire frequency range, DH5α Δ*kch* exhibited higher impedance moduli than the WT. Specifically, the impedance moduli obtained at low frequencies (e.g., 0.25 Hz) are 3-fold higher than those of the WT at the same frequency. This difference in impedance moduli was consistent across the range of applied DC bias voltages (Table S3). This suggests that DH5α provides more efficient transport of cationic ions than DH5α Δ*kch*, which is mediated by the potassium Kch ion channel at low frequencies. The negative capacitance effect was also prominent within the low-frequency region (Figure 3b,d). This result agrees with the Nyquist plots (Figure 3a–d) and the fit parameters from the model, in which the resistance through the *Q* channel and its gating variable were high compared with those of the Kch channel (Tables S1 and S2).

*E. coli* biofilms require higher values of the equivalent circuit elements to model their response to voltage stimulation than neurons, but biofilms contain many small bacterial cells, whereas neuronal experiments are often performed on large individual cells using intracellular electrodes and equivalent electrical circuits for EIS represent extensive physical variables. The resistance *R*_Kch_ across the potassium channel, Kch of *E. coli* biofilms, varied between 140 and 232 ± 1 Ω, while the resistance *R*_*m*_ across its gating variable, *m*, was up to 289 ± 2 Ω (Figure 4f and Table S2). The biofilm inductance had values of 0.94–53.8 ± 0.01 H (Table S3 and Figure S3b). In biofilms, the time constant *τ*_*m*_ = *L*_*m*_/*R*_*m*_ had values between (114 ± 3) × 10^–4^ and (9.6 ± 5.2) × 10^7^ across the range of applied voltages. In contrast, the predicted frequency domain EIS response of single neurons^5^ has typical resistance values, *R*_K_ and *R*_*n*_, ≤0.001 and ≥0.05 Ω, respectively, with the inductance not exceeding 2 H. Such high values of the inductance (negative capacitance) with neurons^4^ and biofilms are characteristic of their nonlinear conductivities, which allow spiking of membrane potentials. The highest value for the time constant, *τ*_*n*_ = *L*_*n*_/*R*_*n*_ of the potassium channel of neurons was approximately 1 × 10^–3^ s.^5^ Neurons can therefore exhibit faster response times than *E. coli* biofilms by an order of magnitude.

Negative capacitance has been extensively linked to ion-channel-mediated conductivity in different types of neuron and is connected to the positive feedback process that causes spiking of membrane potentials.^3−6,37^ The existence of the negative capacitance in both strains of bacterial biofilms indicates the presence of voltage-gated ion channels in *E. coli*. Our previous results show that biofilms of the DH5α Δ*kch* strain engage in ion-channel-mediated membrane potential dynamics when under stress, and distinct changes were observed compared to the biofilm of the wild-type DH5α strain (the second hyperpolarization event in a novel two-spike phenomenon was lost in the Kch mutant).^23^ Our current study also demonstrates that the voltage-gated Kch potassium ion channel plays a major role in the electrical response of *E. coli* biofilms, and the negative capacitance provides a unique signature of metabolically active *E. coli* in biofilms. Our results provide additional evidence of voltage-gating with the Kch potassium channel in *E. coli*. Negative resistance (as opposed to negative capacitance) is also observed in electrochemical impedance spectroscopy with neurons, and it is attributed to the activity of sodium ion channels with both activation and inactivation gating.^3,4^ No negative resistance was observed in the impedance spectra (second and third quadrants) from bacterial biofilms, indicating a lack of sodium-like ion channels.

The presence of the negative capacitance phenomenon in the biofilms of viable bacterial cells (Figure 3a–d) and its absence in the biofilm of nonviable cells (Figure S2d) could be used to quantify the viability of bacterial cells in biofilms. Portable EIS devices could be used with a small nonzero bias voltage to conduct LiveDead assays with bacterial biofilms. The method could be cheap and fast, have high throughput, and have a higher fidelity than previous methods. Specifically, EIS could prove to be less prone to artifacts than standard LiveDead assays using membrane-permeable/-impermeable dyes.^52−54^

We characterized significant negative capacitance in bacterial biofilms at low frequencies, i.e., a neuronal-like inductive effect in bacterial biofilms.^4,5,37^ We believe this provides a unique insight into the electrophysiology of bacterial biofilms and that negative capacitances occur with most bacterial species, e.g., *B. subtilis*,^34^*Pseudomonas aeruginosa*^26^ and *Streptomyces lividans*.^55,24,26,56^ Our electrical circuit models provide robust fits for the experimental data and enable the extraction of key information about the electrophysiology of the biofilm. Crucially the technique does not require Nernstian voltage dyes that can be semiquantitative and involve artifacts.

The human brain is very complex with a network 10^14^ synapses. Challenges therefore occur to build synthetic memristor networks which replicate the spiking responses of brains.^57^ Synthetic biology techniques with bacterial biofilms could provide a basis for the design of simpler, cheaper, environmentally friendly, and scalable bio-inspired devices for neurocomputation.

## Supporting Information Available

Bacterial biofilm culture, electrode preparation, electrochemical impedance spectroscopy experiments, construction of *E. coli* mutants, model fits, the Hodgkin–Huxley model, equivalent circuit models, and comparison with previous EIS measurements with bacteria (PDF)
