# Supplementary material for: Electrical Impedance Spectroscopy with Bacterial Biofilms: Neuronal-like Behavior
Source: Nano Lett. 2024 Feb 6;24(7):2234–41. doi: 10.1021/acs.nanolett.3c04446 (PMC10885197; doi:10.1021/acs.nanolett.3c04446)
Supplement: Supplementary file 1 — nl3c04446_si_001.pdf [file nl3c04446_si_001.pdf]

Supplementary Information

Electrical impedance spectroscopy with bacterial biofilms: neuronal-like behaviour

Emmanuel U. Akabuogu[1,2], Lin Zhang[1], Rok Krašovec[3], Ian S. Roberts[1*], Thomas A. Waigh[2,4+]

*i.s.roberts@manchester.ac.uk

+t.a.waigh@manchester.ac.uk

[1]Division of Infection, Lydia Becker Institute of Immunology and Inflammation, School of Biological Sciences, University of Manchester, Oxford Rd., M13 9PT, UK.

[2]Biological Physics, Department of Physics and Astronomy, University of Manchester, Oxford Rd., Manchester, M13 9PL, UK.

[3]Division of Evolution, Infection and Genomics, School of Biological Sciences, Faculty of Biology, Medicine and Health University of Manchester, M13 9PT, UK.

[4]Photon Science Institute, Alan Turing Building, Oxford Rd., Manchester, M13 9PY, UK.

Bacterial biofilm culture

E. coli biofilm was used as the functional layer material. A day preceding the EIS experiment, the *E. coli* strains, DH5α and a DH5α Δ*kch* mutant were collected from a -80°C glycerol stock and streaked on an agar plate. On the day of the experiment, a single colony of the cell was transferred into 10 ml of Luria Broth (LB) media (Table S4). The glass universal containing the bacterial suspension was incubated overnight at 200 rpm at 37°C. The next day, 100 µl of the inoculum was pipetted into a fresh 10 ml LB and incubated in a shaking incubator at 200 rpm and at 37°C for 4.5 hours or OD_{600} ≈ 0.8. Cells were subsequently adjusted to a starting concentration of 2 x 10^6 CFU/ml for all the strains to ensure the same number of cells in all experiments. CFU/ml was calculated using the plate counting technique. Thereafter, 3 ml of the cell suspension was transferred onto an ITO electrode in the electrochemical cell. Cells were allowed 2 h to enhance cell attachment on the electrode surface at 37°C. Subsequently, 35 ml of fresh sterile LB was added to the cell suspension in the electrochemical cell to ensure proper electrical contact with the reference and counter electrodes. Cells were left to grow statically for 24 hrs at 37°C incubator. The optical density of the cells was measured using a spectrophotometer (JENWAY, Cole-Parmer UK). Each experiment was conducted in fresh electrolyte to eliminate the effect of culture media on the results.

Working electrode cleaning, treatment, and functionalization

The transparent indium tin oxide (ITO) electrode (Sigma Aldrich) was employed as the conducting electrode. The ITO electrode integrates well with bacterial substrates[1,2]. Prior to inoculation of the cell suspension on the ITO electrodes (2 x 2 cm^2), surface pretreatment was carried out to improve bacterial cell adhesion and biofilm cultivation[3,4]. The ITO surface was cleaned by sequentially sonicating for 10 min each in deionized water, acetone, and ethanol. The electrode was dried under nitrogen blow and subsequently treated with UV-ozone for 5 min. Following bacterial cell growth as described above, the biofilm cultivated for 24 hrs on the ITO serves as the electroactive layer material. The ITO electrode was not reused after each experiment.

Device setup and electrochemical impedance spectroscopy

EIS experiments were conducted on a GAMRY potentiostat Reference 600 Plus (GAMRY INSTRUMENTS, UK) using an electrochemical cell with a platinum counter electrode (CH Instruments Inc, US), an ITO working electrode and an Ag/AgCl reference electrode (CH Instruments Inc, US). A schematic diagram of the apparatus is shown in Fig. S1. After the biofilm was grown for 24 h, EIS was performed at a small AC amplitude of 10 mV, a potential of 0 V and a frequency range from 0.2-10^5 Hz. Under the same conditions, EIS analysis was conducted at set times of 16, 18, 20, 22 and 24 h to

monitor the spectral evolution during growth. The same procedure was employed for both *E. coli* DH5α and DH5α Δ*kch* mutant strains. To monitor the EIS of dead cells, the disinfectant Virkon (at a concentration of 1% (1:100)) was added after 24 hrs of cell cultivation. The EIS data was collected 2 to 3 hrs after the addition of the disinfectant. This ensures no viable cells were left in the biofilm.

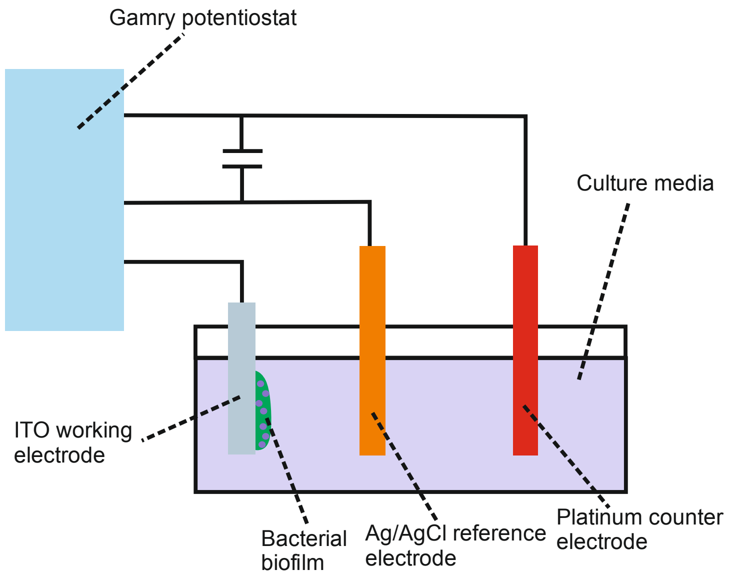

Fig S1. Schematic diagram showing the arrangement of the sample cell for electrical impedance spectroscopy experiments with bacterial biofilms. The biofilm is grown on the ITO working electrode. The reference electrode is Ag/AgCl and the counter electrode is platinum. A Gamry Reference 600 Plus potentiostat is used to meaure the biofilm impedances.

For the antibiotics experiments, we employed rifampicin at 50 μg/ml and streptomycin at 5 μg/ml. Data was collected 3 hrs after the addition of the antibiotics.

To study the effect of varying applied voltages, EIS experiments were performed at a constant small AC amplitude of 10 mV at different DC bias voltages ranging from 0.1 V to 0.8 V. The frequency sweep was maintained from 0.2 to 10^5 Hz. For all experiments, data acquisition, analysis and modeling were done with the GAMRY software (See Table 2).

P1 phage transduction for the construction of *E. coli* DH5α Δ*kch* mutant strain

Using the PCR method, the Kch potassium gene was confirmed in a Keio collection WT strain, *E. coli* K-12 BW25113 strain[5] by amplification using the primers Kch-F (5'-GTGAGTCACTGGGCTACATTCAAAC-3') and Kch-R (5'-CTATTTTTGCGCCGATTCTTTAC-3'). Using the same primers, the Kch gene was not amplified in an *E. coli* K-12 BW25113 Δ*kch*-mutant strain. Subsequently, P1 lysate of the donor strain (*E. coli* K-12 BW25113 Δ*kch*-mutant) was prepared and

transduced into the recipient (*E. coli* DH5α). Once more, the same primers were used to establish the absence of the Kch gene in our new strain, the *E. coli* DH5α Δ*kch* mutant.

Model fit constants

Table S1:

V_{app} (V)	R_Q (Ω)	R_n (Ω)	L_n (H)	$\tau_n = L_n/R_n$ (s)
**0.4**	429 ± 3	14800 ± 100	0.014 ± 0.001	$(94 \pm 7) \times 10^{-8}$
**0.5**	1920 ± 20	25 ± 3	16 ± 2	0.6 ± 0.1
**0.6**	1770 ± 10	260.8 ± 0.6	0.00090 ± 0.00005	$(34 \pm 2) \times 10^{-7}$

Table S1: The parameters extracted from model fits to the EIS spectra of *E. coli* DH5α Δ*kch* (Fig 3d) using the model in Fig 4b. V_{app} is the applied DC bias voltage. R_Q and R_n are resistances across the Q ion channel and its gating variable *n* respectively. Q was used to represent all the voltage-gated ion channels present in the DH5α Δ*kch.* L_n is the inductance across the gating variable *n.* τ_n is the time constant across the RL branch of the circuit, Fig 4b.

Table S2:

V_{app} (V)	R_{Kch} (Ω)	R_m (Ω)	L_m (H)	$\tau_m = L_m/R_m$(s)
**0.4**	140 ± 1	$(6 \pm 3) \times 10^{-7}$	53.8 ± 0.1	$(10 \pm 5) \times 10^{7}$
**0.5**	181 ± 1	125.4 ± 0.8	0.94 ± 0.01	$(750 \pm 9) \times 10^{-5}$
**0.6**	211 ± 3	220 ± 1	2.68 ± 0.02	$(121 \pm 1) \times 10^{-4}$
**0.7**	232 ± 1	289 ± 2	3.2 ± 0.1	$(111 \pm 3) \times 10^{-4}$

Table S2: Parameters extracted from the fits to the EIS spectra of *E. coli* DH5α (Fig 3b) using the model in Fig 4c. V_{app} is the applied DC bias voltage. R_{Kch} and R_m are resistances across the Kch ion channel and its gating variable *m* respectively. L_m is the inductance across the gating variable *m.* τ_m is the time constant across the RL branch of the circuit, Fig 4c.

Table S3:

Strain	$\lvert Z\rvert_{freq = 0.25\,Hz}$ at $V_{app} = 0.4\,V$	$\lvert Z\rvert_{freq = 0.25\,Hz}$ at $V_{app} = 0.5\,V$	$\lvert Z\rvert_{freq = 0.25\,Hz}$ at $V_{app} = 0.6\,V$	$\lvert Z\rvert_{freq = 0.25\,Hz}$ at $V_{app} = 0.7\,V$
**DH5α Δ*kch***	508 ± 4	293 ± 2	258 ± 1	–
**DH5α**	188 ± 2	109.4 ± 0.2	92.4 ± 0.1	86.6 ± 0.3

Table S3: The impedance modulus at low frequency for both strains of *E. coli* biofilm for the range of applied DC bias voltages. Data was obtained at 0.25 Hz across the range of applied voltages with Bode plots similar to Fig 4h. The frequency of 0.25 Hz is equivalent to a log Frequency value of -0.6. $\lvert Z\rvert_{freq}$ represents the modulus of the impedance at a specific frequency. V_{app} is the applied DC bias voltage.

Table S4:

Media	Recipe	
**Luria Broth (LB)**	10g/l NaCl, 5g/l yeast extract, 10g/l Tryptone. Distilled water to 1L	
**LB agar**	10g/l NaCl, 5g/l yeast extract, 10g/l Tryptone, 15 g/l agar.	
**Experimental Models: Strains**		
***E. coli* DH5α**	Ian Robert's lab	
***E. coli* DH5α (Δ*kch*)**	This study	
***E. coli* BW25113 (JW1242-1)**	Keio Collection [8]	
***E. coli* BW25113 (Δ*kch*)**	Keio Collection [8]	
**Software and Data Analysis**		
**Python (Anaconda)**	Python	https://anaconda.org/ContinuumIO
**GAMRY**	GAMRY	https://www.gamry.com/support-2/software/
**Electrochemical cell and Electrodes**		
**Ag/AgCl**	CH Instruments Inc, US	
**Platinum (Pt)**	CH Instruments Inc, US	
**Transparent Indium Tin oxide (ITO)**	Sigma-Aldrich	
**Electrochemical cell**	GaossUnion, China	
**GAMRY Potentiostat**	GAMRY Instruments Inc, US	

Table S3: Media recipe, bacterial strains, software and EIS components used in experiments on *E. coli* biofilms.

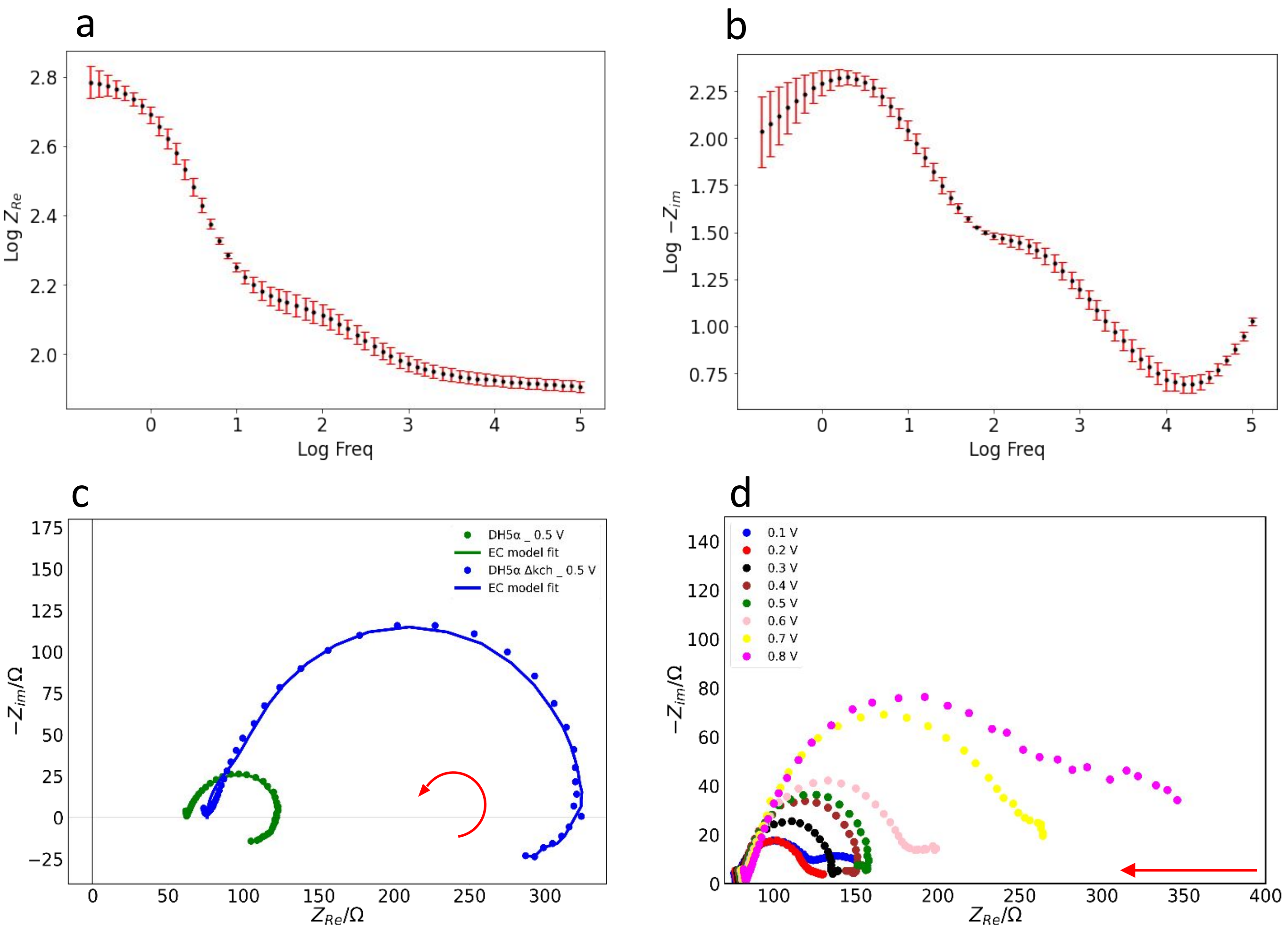

Fig S2: (a) The real impedance (Z_{Re}) plotted as a function of frequency from experiments with the wildtype DH5α biofilms (Fig 1b) (mean ± SD for three repeats). b) Imaginary impedance $(-Z_{Im})$ as a function of frequency from the wildtype DH5α biofilms (Fig 1b). c) Representative data showing that our proposed minimal equivalent circuit provides a good fit to experimental data with the imaginary $(-Z_{Im})$ plotted as a function of the real impedance (Z_{Re}). The equivalent circuits of Figures 4b and 4c were used for the mutant and wildtype respectively. The solid lines are model fits and the dots are the experimental data at the applied DC bias voltage of 0.5 V. The plot also compares differences in the impedance arcs at similar DC bias voltages for both strains. Each point shows a separate frequency. d) EIS data for DH5α biofilm with no viable cells (subjected to Virkon for 2 hours) for different DC bias voltages showing the complex impedance $(-Z_{Im})$ as a function of the real impedance (Z_{Re}). Each point shows a separate frequency. Red arrows show the direction of increasing frequency.

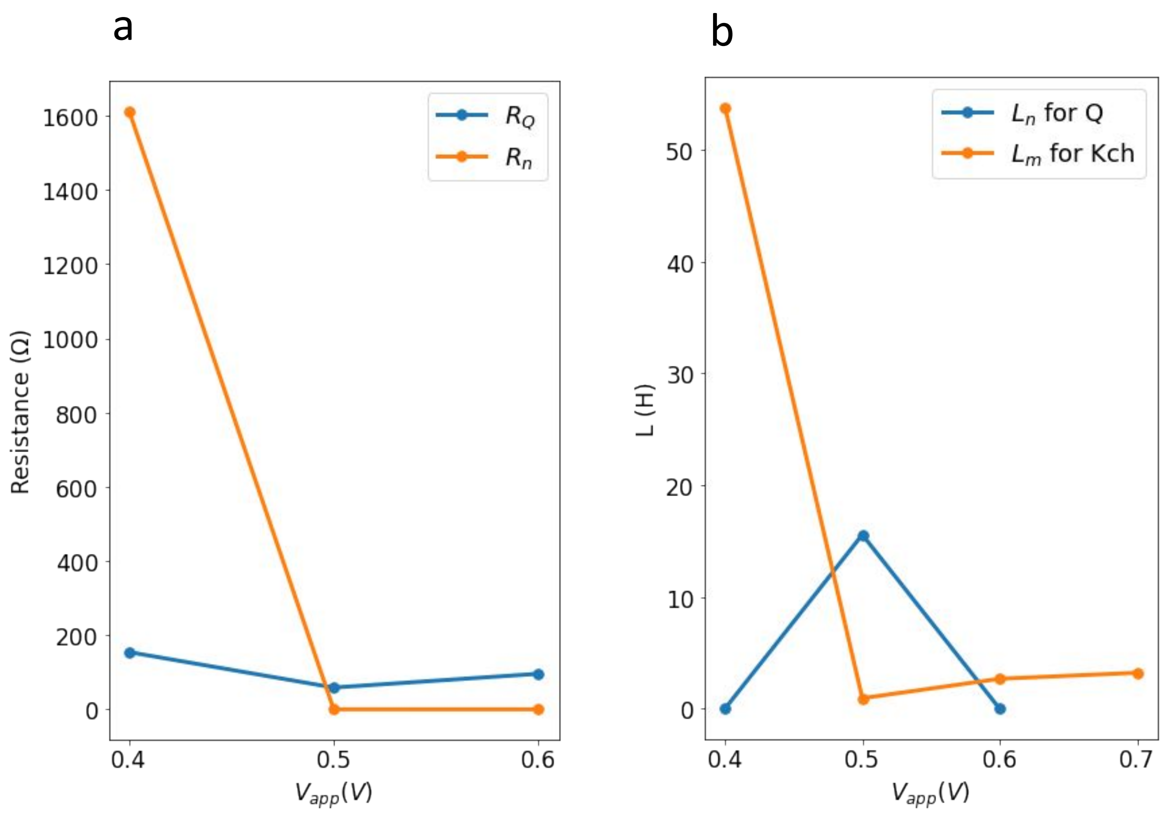

Fig S3: a) Values of the resistance for the Kch mutant as a function of the DC bias voltage (V_{app}) from the EIS data shown in Fig 3d obtained using the complete equivalent circuit model shown in Fig 4c. R_Q and R_n are resistances across the Q ion channel and its gating variable *n* respectively. b) Values of the inductances (L) as a function of the DC bias voltage (V_{app}) for both *E. coli* biofilm strains using their respective equivalent circuits models. L_n and L_m are the inductances across the gating variables *n* and *m* of the Q and Kch ion channels respectively.

Mathematical model of the frequency domain impedance response of *E. coli* biofilms

A single channel time-dependent Hodgkin-Huxley model[6] was developed to explain the stress-dependent electrical signalling in *B. subtilis* biofilms[7]. We extended this model to understand the two-channel mediated membrane potential dynamics in *E. coli* biofilms in response to blue light stress[8]. In the current work, we used the same two-channel Hodgkin-Huxley model as our previous study in *E. coli*[8] to understand electrical impedance spectroscopy measurements with *E. coli* biofilms.

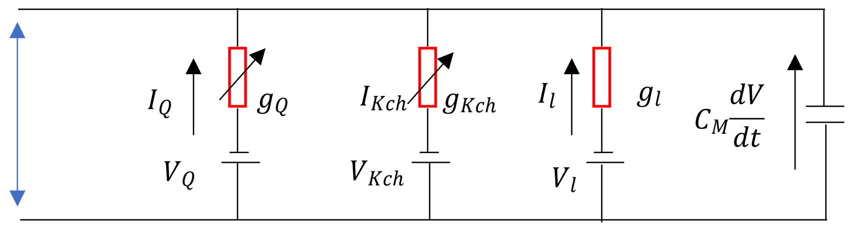

Fig S4: A Hodgkin-Huxley equivalent circuit model for the time-dependent conductance of *E. coli* biofilms[8]. g_Q, g_{Kch} and g_l represent the conductance of the, Q, Kch and the leak channel l respectively. V_q , V_{Kch} and V_l represents the Nernst potentials for the Q, Kch and leak ions respectively. I_Q, I_{Kch} and I_l are currents through the Q, Kch and leak channels respectively. $C_M(^{dV}/dt)$ represents the capacitive current across the membrane.

Fig S4 shows the equivalent circuit used in the HH model of *E. coli* biofilms in which the bacteria are modelled with two ion channels Kch and Q. The currents through the ionic channels (the equivalent resistors) and the cell membrane (the equivalent capacitor) in Fig S4 are

$$I_c = C_M \frac{dV_M}{dt}, \quad 1$$

$$I_Q = g_Q(V_M - V_Q), \quad 2$$

$$I_{Kch} = g_{Kch}(V_M - V_{Kch}), \quad 3$$

and

$$I_l = g_l(V_M - V_l). \quad 4$$

where $g_j = {}^{1}/_{R_j}$ is the conductance of the j ion channel. Thus g_Q, g_{Kch} and g_l are the conductances of the, Q, Kch and leak channel l respectively. V_q , V_{Kch} and V_l represent the Nernst potentials for the Q, Kch and leak ions respectively. Q represents all voltage-gated channels other than the Kch ion channel which are in the DH5α Δ*kch* mutant. V_M is the resting Nernst potential.

We assumed that both positively charged ion channels, Q and Kch, have four subunits which are their four activation gates. The variable *n* represents the gating variable for the Q channels and *m* is the gating variable for Kch channel. We can therefore rewrite eqns 2, 3 and 4 as

$$I_Q = \frac{1}{R_Q} n^4 (V_M - V_Q) \,, \qquad 5$$

$$I_{Kch} = \frac{1}{R_{Kch}} m^4 (V_M - V_{Kch}) \,, \qquad 6$$

$$I_l = \frac{1}{R_l} (V_M - V_l) \,. \qquad 7$$

The fraction of time each channel is open can then be represented by

$$\frac{dn}{dt} = \alpha_n(S)(1-n) - \beta_n \,, \qquad 8$$

and

$$\frac{dm}{dt} = \alpha_m(S)(1-m) - \beta_m \,, \qquad 9$$

for the Q and Kch channels respectively. S represents the stress level[8–10], e.g. due to voltage stimulation or light stress. β_n and β_m stand for the rate at which the open channels close and they are stress-dependent. α_n and α_m are the rates at which closed channels open and they are stress-dependent. R_Q, R_{Kch}, and R_l are resistances across the Q, Kch and l respectively.

Busquert et al. provided a small perturbation AC model for the frequency domain impedance response of the standard HH model for a neuron (with both potassium and sodium ion channels) which applies to each branch of the neuronal equivalent circuit[11]. Based on our experimental observations, we therefore extended this version of the HH model to develop a minimal model for the frequency domain impedance response of *E. coli* biofilms under small AC perturbations i.e. we include two

potassium-type ion channels because negative capacitances are observed, but no negative resistances (no sodium-type ion channels are observed).

To model AC perturbations of the HH model, a Laplace transform of eqns 1, 5, 6 and 7 yields

$$\tilde{I}_C = sC_M\tilde{V}_M\,, \qquad 10$$

$$\tilde{I}_Q = \frac{1}{R_Q}4\bar{n}^3(\bar{V}_M - V_Q)\tilde{n} + \frac{1}{R_Q}\bar{n}^4\tilde{V}_M\,, \qquad 11$$

$$\tilde{I}_{Kch} = \frac{1}{R_{Kch}}4\bar{m}^3(\bar{V}_M - V_{Kch})\tilde{m} + \frac{1}{R_{Kch}}\bar{m}^4\tilde{V}_M\,, \qquad 12$$

$$\tilde{I}_l = \frac{1}{R_l}\tilde{V}_M, \qquad 13$$

where $\tilde{I}_C$, $\tilde{I}_Q$ and $\tilde{I}_{Kch}$ are the current across the membrane, the Q, Kch and l respectively. $\tilde{V}_M$ is the resting Nernst potential. The Also $\tilde{n}$, $\tilde{m}$ are the perturbed gating variables. The tilde sign (~) represents the small perturbation value. $\bar{n}$ and $\bar{m}$ represent the value of the gating variables at steady state. $\bar{V}_M$ is the value of the Nernst potential at a steady state. The overbar (¯) represents the value at a steady state.

The Laplace transform of the gating variables *n* and *m* which are defined eqns 8, 9 and which also appear in eqns 11, 12 yields

$$s\tilde{n} = \left[\frac{\partial\bar{\alpha}_n}{\partial V_M}(1-\bar{n}) - \frac{\partial\bar{\beta}_n}{\partial V_M}\bar{n}\right]\tilde{V}_M - (\bar{\alpha}_n + \bar{\beta}_n)\tilde{n} \qquad 14$$

$$s\tilde{m} = \left[\frac{\partial\bar{\alpha}_m}{\partial V_M}(1-\bar{m}) - \frac{\partial\bar{\beta}_m}{\partial V_M}\bar{m}\right]\tilde{V}_M - (\bar{\alpha}_m + \bar{\beta}_m)\tilde{m} \qquad 15$$

The impedance *Z* for the frequency domain response is

$$Z = \frac{\tilde{V}_M}{\tilde{I}_M} = \frac{\tilde{V}_M}{\tilde{I}_C + \tilde{I}_Q + \tilde{I}_{Kch} + \tilde{I}_l}. \qquad 16$$

Substituting the eqns 10-15 into 16, we obtain

$$Z = \left[sC_M + \frac{1}{R_Q} + \frac{1}{R_n + sL_n} + \frac{1}{R_{Kch}} + \frac{1}{R_m + sL_m}\right]^{-1} \qquad 17$$

where *s* is the Laplace frequency, ω is the frequency and $s = i\omega$. The overbar denotes the values at a steady state while the tilde represents the small perturbation. R_n and R_m denote the resistances through the gating variable *n* and *m* respectively. L_n and L_m are the inductance across the gating variable *n* and *m* respectively.

Eqn 17 allows the frequency domain response of the electrical equivalent circuit Fig 4c of the *E. coli* biofilm to be calculated.

Each circuit element which depends on the voltage can then be deduced as stated below:

$$R_Q(\bar{V}_M) = \frac{R_Q}{\bar{n}^4} \qquad 18$$

$$R_n(\bar{V}_M) = \frac{R_Q}{4\bar{n}^3(\bar{V}_M - V_Q)\tau_n\left[\frac{\partial\bar{\alpha}_n}{\partial V_M}(1-n) - \frac{\partial\bar{\beta}_n}{\partial V_M}\bar{n}\right]} \qquad 19$$

$$L_n(\bar{V}_M) = R_n\tau_n \qquad 18$$

$$R_{Kch}(\bar{V}_M) = \frac{R_{Kch}}{\bar{m}^4} \qquad 20$$

$$R_m(\bar{V}_M) = \frac{R_{Kch}}{4\bar{m}^3(\bar{V}_M - V_{Kch})\tau_m\left[\frac{\partial\bar{\alpha}_m}{\partial V_M}(1-m) - \frac{\partial\bar{\beta}_m}{\partial V_M}\bar{m}\right]} \qquad 21$$

$$L_m(\bar{V}_M) = R_m\tau_m \qquad 22$$

where τ_n and τ_m are the relaxation time constants for the gate variables, *n* and *m* respectively. $\overline{\alpha}_n$ and $\overline{\beta}_n$ are the values of the gating variable *n* at steady state. $\overline{\alpha}_m$ and $\overline{\beta}_m$ are the values of the gating variable *m* at steady state.

Other considerations for the development of the equivalent circuit model:

a) The Gamry software provides similar fits to those using analytic expressions explicitly implemented in Python i.e. using the Hodgkin-Huxley model described above.

b) The negative capacitances have values that are a robust feature of all reasonable models used to describe the data (the key experimental finding) and correspond to a clear feature in the data (an arc into the 4th quadrant of the impedance data).

c) Simultaneous use of wild type and Kch knock down mutants allows the ambiguity in the modelling to be reduced. Availability of more ion channel mutants would provide additional sensitivity in determining the origins of the currents.

d) Two of the resistor values in the parallel branch are coupled in the model (R_Q and R_l). They could be lumped together, but they are considered separately so that an identical equivalent circuit is used in both the current manuscript and our previous *eLife* article[8]. The other branches of the model are decoupled in the model due to their different frequency dependencies.

e) Our equivalent circuit model is for the entire biofilm (in general it is common in electrical impedance spectroscopy to create such equivalent circuits for entire films). It would be interesting to explicitly incorporate the activity of ion channels for the response of each individual cell and then combine them with coupling effects to describe the entire biofilm. This has not yet been done for bacterial cells in the literature. A promising method would be to adapt the agent based model presented in our previous *eLife* article[8] to predict impedance spectra. To our knowledge this has not previously been achieved in the literature on electrical impedance spectroscopy from any cells or colloids, although there has been some success using finite element models on tissue.

f) The contact resistance R_{ct} describes the transfer of charge from the electrodes to the film. In the specific case of biofilms, it is expected to be due to a complex range of effects including adsorption to the electrodes by extracellular polymeric substance, adhesion complexes on the membranes of the bacteria (e.g. pili) and electrophysiological effects of the bacteria membrane potential (e.g. voltage-gated ion channels).

g) All the bacteria biofilms studied have a very similar number of bacterial cells. It is expected that the DC bias voltage required to observe negative capacitance will scale with the number and type of

bacterial cells. Furthermore, the DC bias voltage is expected to scale with the logarithm of the potassium concentration within the growth media (it is a Nernstian membrane potential).

h) The constant phase element (CPE) is commonly invoked in electrical impedance spectroscopy experiments to account for a power law background. Its origins are still debated for inorganic materials, although the non-integer power-law scaling indicates it could be a fractal effect e.g. with biofilms it could be due to the fractal structure of the extracellular polymeric substance or the fractal (anomalous) dynamics of the ions in the biofilm.

Comparison with previous impedance spectroscopy measurements from bacteria and bacterial biofilms

Why have negative capacitances not been previously observed from bacteria or bacterial biofilms?

We believe a principal issue is the small value of the impedance for single bacteria. Much higher signal to noise can be achieved using large numbers of bacteria in bacterial biofilms. Thus biofilms provide higher resolution electrical impedance spectra.

Furthermore, the culture of biofilms is a specialist area and they were only first recognized as separate well defined states of bacterial physiology in the late 1970s (Costerton coined the word in 1978). This will have obstructed older studies.

A critical requirement to observe the negative capacitance is the application of a DC bias voltage. Many of the previous studies have neglected to explore the phenomenon (we were motivated by the recent article of Bou et al[11] in 2021) and it could require bespoke instrumentation to be developed in some cases (we were fortunate that is was a standard feature on the commercial Gamry apparatus).

Below we consider some key previous articles in the EIS literature:

Ward, A. C., Connolly, P. & Tucker, N. P. Pseudomonas aeruginosa Can Be Detected in a Polymicrobial Competition Model Using Impedance Spectroscopy with a Novel Biosensor. *PLoS One* **9**, 91732 (2014).

Analysis: bespoke apparatus is used to consider *P. aeruginosa* biofilms EIS. The authors do not apply a DC bias voltage. They stress the importance of electroactive metabolites and the adhesion to the electrodes, but miss the effect of bacterial spiking potentials and voltage-gated ion channels.

Kim, T., Kang, J., Lee, J. H. & Yoon, J. Influence of attached bacteria and biofilm on double-layer capacitance during biofilm monitoring by electrochemical impedance spectroscopy. *Water Res* **45**, 4615–4622 (2011).

Analysis: a commercial CH instrument instrument was used for EIS. A constant phase element (CPE) was needed to describe the data, similar to the current study. The authors do not apply a DC bias voltage. The effect of bacterial spiking potentials and voltage-gated ion channels is neglected.

Barreiros dos Santos, M. *et al.* Highly sensitive detection of pathogen Escherichia coli O157:H7 by electrochemical impedance spectroscopy. *Biosens Bioelectron* **45**, 174–180 (2013).

Analysis: a Princeton Applied Research VMP2 multipotentiostat was used for EIS. The device was optimised for low concentrations of pathogenic *E. coli*. A constant phase element was used to analyse the data. The authors do not apply a DC bias voltage. The effect of bacterial spiking potentials and voltage-gated ion channelsis is neglected.

Yang, Y. et al Measurement of the low-frequency charge noise of bacteria. Physical Review E 105, 064413 (2022).

Analysis: the authors develop a bespoke microfluidic EIS device with a lock-in amplifier. They assume a simple equivalent circuit to describe single bacteria and do not measure the phase information directly to produce the true EIS measurements required to observe negative capacitances.

REFERENCES

1. Jain, A., Gazzola, G., Panzera, A., Zanoni, M. & Marsili, E. Visible spectroelectrochemical characterization of Geobacter sulfurreducens biofilms on optically transparent indium tin oxide electrode. *Electrochim Acta* **56**, 10776–10785 (2011).
2. Barreiros dos Santos, M. *et al.* Label-free ITO-based immunosensor for the detection of very low concentrations of pathogenic bacteria. *Bioelectrochemistry* **101**, 146–152 (2015).
3. Schmidt, I. *et al.* Gold-modified indium tin oxide as a transparent window in optoelectronic diagnostics of electrochemically active biofilms. *Biosens Bioelectron* **94**, 74–80 (2017).
4. Virdis, B. *et al.* Analysis of electron transfer dynamics in mixed community electroactive microbial biofilms. *RSC Adv* **6**, 3650–3660 (2016).
5. Baba, T. *et al.* Construction of Escherichia coli K-12 in-frame, single-gene knockout mutants: the Keio collection. *Mol Syst Biol* **2**, 1–10 (2006).
6. Hodgkin, A. L. & Huxley, A. F. A quantitative description of membrane current and its application to conduction and excitation in nerve. *J Physiol* **117**, 500–544 (1952).

7. Prindle, A. *et al.* Ion channels enable electrical communication in bacterial communities. *Nature* **527**, 59–63 (2015).

8. Akabuogu, E. U., Martorelli, V., Krašovec, R., Roberts, I. S. & Waigh, T. A. Emergence of ion-channel mediated electrical oscillations in E. coli biofilms. *bioRxiv* 2022.11.09.515771 (2022) doi:10.1101/2022.11.09.515771.

9. Blee, J. A., Roberts, I. S. & Waigh, T. A. Spatial propagation of electrical signals in circular biofilms: A combined experimental and agent-based fire-diffuse-fire study. *Phys Rev E* **100**, 1–9 (2019).

10. Blee, J. A., Roberts, I. S. & Waigh, T. A. Membrane potentials, oxidative stress and the dispersal response of bacterial biofilms to 405 nm light. *Phys Biol* **17(3)**, 1–10 (2020).

11. Bou, A. & Bisquert, J. Impedance Spectroscopy Dynamics of Biological Neural Elements: From Memristors to Neurons and Synapses. *Journal of Physical Chemistry B* **125**, 9934–9949 (2021).

12. Cole, K. S. Rectification and inductance in the squid giant axon. *J Gen Physiol* **25**, 29 (1941).
